# Ultrasonographic Elastography of the Spleen for Diagnosing Neoplastic Myeloproliferation: Identifying the Most Promising Methods—A Systematic Review

**DOI:** 10.3390/jcm14155400

**Published:** 2025-07-31

**Authors:** Mateusz Bilski, Marta Sobas, Anna Zimny

**Affiliations:** 1 Department of General and Interventional Radiology and Neuroradiology, Wrocław Medical University, 50-367 Wroclaw, Poland; 2 Department of Hematology, Collegium Medicum in Bydgoszcz, Nicolaus Copernicus University in Toruń, 85-067 Bydgoszcz, Poland

**Keywords:** ultrasound diagnosis, spleen stiffness, myeloproliferative neoplasms

## Abstract

**Background**: The relationship between spleen and bone marrow stiffness, and other features of abnormal myeloproliferation has long been described. However, the scientific knowledge in this area remains very superficial. This review evaluated the diagnostic effectiveness of various ultrasound (US) methods in the assessment of neoplastic myeloproliferation using spleen stiffness measurement (SSM). **Aim**: To explore the diagnostic accuracy of US techniques in assessing spleen stiffness, determining which of them may be suitable for the diagnosis of myeloproliferative diseases in adults. **Methods**: The review included original retrospective or prospective studies published in the last five years (2019–2024) in peer-reviewed medical journals that reported receiver operating characteristics (ROCs) for SSM and the articles concerning the relation between SSM values and neoplastic myeloproliferation. The studies were identified through PubMed searches on 1 July and 1 December 2024. Quality was assessed using the QUADAS-2 tool. Results were tabulated according to the diagnostic method separately for myeloproliferative neoplasms (MNs) and for other clinical findings. **Results**: The review included 52 studies providing ROCs for SSM or compatibility between operators, and five studies covering the relation between SSM values and MNs. **Conclusions**: Acoustic radiation force impulse (ARFI), two-dimensional shear wave elastography (2D-SWE), transient elastography (TE), and point shear wave elastography (p-SWE) are promising methods for measuring SSM that can be incorporated into the diagnosis, screening, and monitoring system in MNs.

## 1. Introduction

Myeloproliferative neoplasms (MNs), including especially chronic myeloid leukemia (CML), polycythemia vera (PV), essential thrombocythemia (ETH), and primary myelofibrosis (MYF) are disorders characterized by pathologic proliferation of hematopoietic stem cells [1,2,3,4]. Epidemiology of MNs is obviously increasing [5,6,7]. These diseases often cause changes (or even lesions) in the spleen [8,9], such as pathomorphological alterations resulting primarily from extramedullary hematopoiesis, including splenomegaly (often massive), expansion of the red pulp with trilineage hematopoietic proliferation, fibrotic changes, and splenic infarcts. In imaging diagnostics—especially ultrasonography—the spleen typically appears enlarged with heterogeneous echotexture and possible hypoechoic or hyperechoic lesions, while CT can reveal hypoattenuating infarcted or fibrotic areas, and MRI shows these changes as hypointense zones on T1- and T2-weighted images with potential enhancement abnormalities on contrast studies.

Elastography is a group of ultrasound-based (US) techniques that assesses the mechanical properties of tissues, particularly their stiffness and elasticity.

Acoustic radiation force impulse (ARFI) imaging generates a mechanical excitation across the examined tissue structures via focused acoustic energy, measuring shear wave speed as a surrogate for stiffness. Unlike point or 2D elastography, ARFI does not rely on real-time color mapping and provides localized, qualitative, or semi-quantitative stiffness estimates displayed directly on the B-mode image (i.e., standard grayscale ultrasound image showing tissue structure) [10,11,12].

Point Shear Wave Elastography (p-SWE) measures shear wave velocity (SWV) at a localized region of interest and outputs quantitative values [13,14,15].

Two-Dimensional Shear Wave Elastography (2D-SWE) enables real-time imaging of tissue stiffness across a wider field of view, providing color pictures (so-called elastograms) with quantitative measurements [16,17,18,19].

Supersonic Shear Imaging (SSI) is a proprietary form of 2D-SWE that uses multiple supersonic push pulses to generate shear waves, enabling ultrafast imaging (>5000 frames per second) and high-resolution quantitative elastograms across large tissue areas [20,21,22].

Transient Elastography (TE) uses a mechanical vibrator to generate a low-frequency shear wave and a one-dimensional ultrasound transducer to detect its propagation speed. Unlike other techniques, TE does not produce real-time B-mode images and provides only a single averaged stiffness value from a predefined volume [23,24,25].

Vibration-Controlled Transient Elastography (VCTE) uses a mechanical vibrator to generate low-frequency shear waves (typically 50 or 100 Hz), with ultrasound tracking their speed to quantify tissue stiffness [26,27].

Impulse Elastography (e.g., FibroScan^®^) refers specifically to the implementation of VCTE technology by Echosens^®^, combining axial mechanical impulses and A-mode ultrasound tracking in a standardized, operator-independent system for liver and spleen stiffness measurement [28,29].

Endoscopic Ultrasound Elastography (EUS-elastography) delivers elastographic assessment via an endoscopic ultrasound probe, enabling stiffness evaluation of deep structures like the spleen and portal system [30,31].

Across the above modalities, the main parameters assessed include SWV (in meters per second), reflecting the speed of propagation of mechanical waves through tissue, and elastic modulus or stiffness (in kilopascals), derived from SWV and tissue density, indicating tissue rigidity.

In the spleen, elastographic findings have been correlated with several pathophysiological changes. Increased spleen stiffness is associated with extramedullary hematopoiesis, congestion, fibrotic transformation, and architectural disruption—all commonly observed in MNs and in portal hypertension-related conditions [32,33,34,35].

It has long been known that evaluation of the spleen can open the way to effective, non-invasive diagnosis and monitoring of these diseases. Conventional US, including B-mode and color Doppler, remains an essential tool routinely used by hematologists to assess spleen size and to exclude focal lesions during follow-up. In the past decade, however, TE and other elastographic techniques have emerged as valuable complementary methods. Elevated SSM values have also been linked to worse clinical outcomes, such as higher degrees of bone marrow fibrosis and the presence of high-risk esophageal varices [10,36,37,38,39].

However, applying for SSM entails particular challenges. The spleen is smaller and more deeply located compared to the liver, with its upper part often obscured by ribs and intestinal gas. Its relatively softer baseline stiffness and smaller volume mean that measurements are more prone to error, mistake, bias, and operator variability as well [40,41].

Moreover, current stiffness measurement standards established for liver assessment cannot be simply transferred to spleen, as SSM depends not only on fibrotic structures distribution (like in the case of liver), but also heavily on vascular factors, like portal tension value. Differences in the SSM thresholds for normal versus pathological spleen vary depending on techniques and devices [40,41].

Recent technological advances have enhanced the diagnostic accuracy of spleen stiffness measurement. Nonetheless, reproducibility (often measured via intraclass correlation coefficients, ICCs) varies depending on operator experience, patient body habitus, and spleen size.

Since the decreases in splenic stiffness measurement (SSM) and splenic sound speed and spleen size can be observed during treatment in MNs, the added value of these measurements remains unclear [10]. It has been hypothesized that the use of non-invasive diagnostic methods such as, for example, US, could help in the detection of patients with MNs through diagnostic screening, as well as non-invasive monitoring of their condition without the use of invasive methods such as bone marrow biopsy [36,37,38,39].

Slot et al. presented an extensive systematic review in which they demonstrated the poor state of scientific knowledge and serious shortcomings in existing studies regarding different imaging methods (bone marrow, liver, and spleen imaging) for the diagnosis of MNs [36]. Although they highlighted the need for future research, including elastographic SSM, there is no widespread response in the literature.

There is undoubtedly a wide scientific knowledge about SSM, especially gained from research on portal hypertension and esophageal varices (EVs). The research has established that spleen SSM reliably correlates with portal pressure, and therefore predicts the presence and severity of varices, and may serve as a non-invasive marker of clinically significant portal hypertension (CSPH). There still exists a need to determine whether this well-known imaging parameter could be used in a diagnostic algorithm for MNs, even one resembling the Baveno criteria used in the gastroenterological applications. There is also a need to determine which ultrasound methods should be studied for this purpose.

To assess these goals, this article reviews the current state of knowledge on the diagnostic performance of SSM in various clinical conditions, and compares these findings with the limited available data on SSM in patients with MNs.

Accordingly, this paper first synthesizes the abundant evidence on SSM in non-neoplastic conditions— especially PH and EVs—before addressing the limited data available for MNs, identifying and discussing the existing gaps as well as defining priorities for future research.

## 2. Materials and Methods

This review followed the PRISMA 2020 guidelines [42,43].

No review protocol had been formally established or registered for this research.

Articles highlighting recent advancements in spleen ultrasound diagnostics were reviewed.

Studies that provided data allowing comparison of different ultrasound techniques for assessing spleen stiffness were included. Eligible articles comprised original retrospective or prospective studies with randomized or consecutive groups. Articles containing data on the association between the SSM value measured by a specific US method and the myeloproliferative condition were included to answer the first research question.

Articles offering area under the receiver operating characteristic curve (AUROC) calculations, an internationally recognized standard for comparing diagnostic imaging methods, were included to answer the second research question.

Articles that lacked key parameter specifications or sufficient details for reliability analysis (e.g., unclear study flow), publications outside peer-reviewed medical journals, studies solely involving pediatric populations, and review articles were excluded. Data were organized in tables and synthesized based on examined pathology, diagnostic modality, and technique.

The PubMed database was searched on 7 July 2024 and again on 1 December 2024 (iteration). Filters included the last 5 years (2019–2024), English abstract, and preprints excluded.

The search terms consisted of the following keywords/phrases (coexistent): ((“spleen stiffness”[tiab] OR SSM[tiab]) AND (AUROC[tiab] OR AUC[tiab] OR “receiver operating characteristic”[tiab] OR curve[tiab])) OR ((myeloproliferative[tiab] OR myeloproliferation[tiab]) AND ultrasound[tiab]).

Articles meeting the criteria were identified by double-screening titles and abstracts, excluding duplicates in the process. After retrieval, full texts were reviewed against the inclusion and exclusion criteria by two independent researchers without the use of automation. Each pair of researchers reviewed results for different conditions.

Data extraction was performed directly from the texts by two independent researchers without automation. Extracted values were assessed for the likelihood of obvious errors (e.g., mean outside confidence interval). Data validation was performed separately by two scientists for all results.

The reported outcome data included AUROC values or ICC values, for the US modality or method, and patient groups. The results were organized into tables based on the purpose of SSM employment. For each study, a qualitative interpretation of the obtained AUROC values was performed to compare the effectiveness of the methods.

The risk of bias was independently investigated by two scientists. As part of the initial evaluation, a flowchart was created for each article and studies were categorized as retrospective and prospective. The QUADAS-2 tool [43] was used for this assessment.

This assessment was carried out in four domains: patient selection, index test, reference standard, and flow/timing. Yes/no questions derived from the tool were used, with applicability assessed for the first three domains.

Notably, the QUADAS-2 tool is a two-stage assessment system, categorizing criteria as either un-met or uncertain, without an option for “some concerns.”

To answer the first research question, arbitrary effect measures used by the authors were adopted. In the case of the second research question, the primary outcome measure in the synthesis was the AUROC, with the effect measured as the difference between the AUROC values. Data tabulation was used as the synthesis method. For studies that included MNs, where AUROC was not always available, we included outcome measures as reported by the authors to avoid a loss of valuable data.

We did not perform additional searches for inter-operator compatibility (ICC). However, where ICC is given we have taken it into account. The separate consideration of this measure is due to the fact that the diagnosis of the spleen is largely discretionary, especially in patients without splenomegaly. It is therefore necessary to also take into account the extent to which a given diagnostic method is in fact clinically objective.

The results were divided according to the analyzed techniques, and the descriptive statistics for the tables were determined.

The methods used to assess certainty in the body of evidence included the QUADAS-2 tool for evaluating the risk of bias, inclusion and exclusion criteria, careful data extraction and verification for accuracy, and the synthesis of data focusing on reported AUROC values.

No meta-analysis was performed due to substantial heterogeneity of setting across studies (populations, disease severity, elastographic techniques). In accordance with PRISMA Items 13d and 13e, this decision was made a priori. Subgroup and sensitivity analyses were not conducted due to the limited number of studies within comparable subgroups (this is because the number of results for the MNs subgroup would be too small to be of evidential significance).

## 3. Results

**Figure 1:**
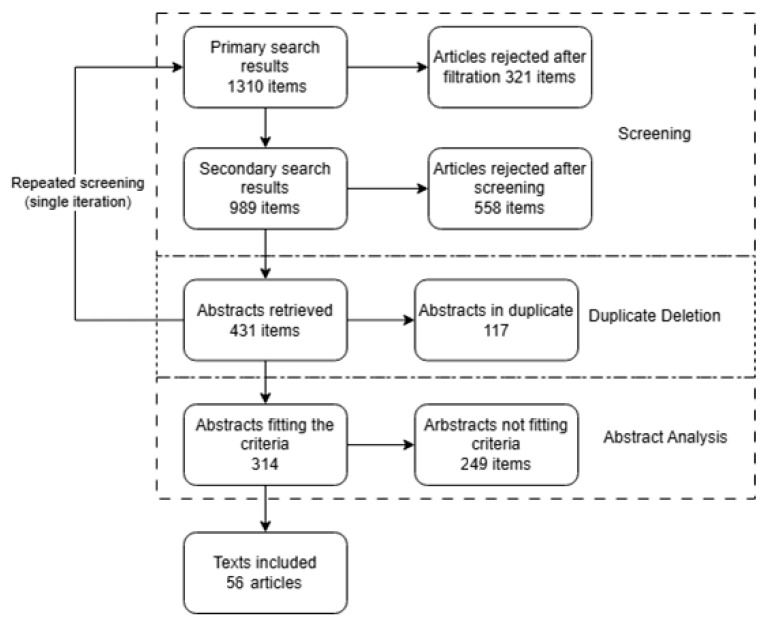
Study flow diagram.

A total of 1310 results were screened, and 47 texts were included (Figure 1).

At the title- and keyword-screening stage, articles whose content clearly failed to meet the predefined eligibility criteria were excluded. The most frequent reasons were the absence of original quantitative data (e.g., no numerical results in the accessible version, or the article was purely narrative/review) and studies conducted exclusively in pediatric populations. Because the search strategy employed two keyword combinations and each search was repeated to assure no material was omitted, the final list was checked for duplicates, with 117 records discarded on this basis.

It should be emphasized that several manuscripts excluded at the abstract-review stage for not meeting the inclusion/exclusion criteria may nonetheless contain clinically relevant information and are therefore referenced below.

The following studies were excluded.

A well-designed study by Xu HF et al. was in Chinese with no relevant data in the English abstract [44]. Although we did not exclude non-English articles by design, we were unable to translate this article properly. Ferreira-Silva provided AUROC values for the SSM to liver stiffness ratio but not for SSM alone [45]. Nababan et al. did not provide AUROC values for SSM but reported specificity and sensitivity [46]. They evaluated VCTE (by FibroScan) efficacy in high-risk esophageal varices [HREVs] among cirrhotic patients in Indonesia and noted a sensitivity of 98.1% (89.7–99.9%), 86.5% (74.2–94.4%), and 50% (35.8–64.2%) for 20 kPa SSM, 40 kPa SSM, and 70 kPa SSM, respectively, as well as a specificity of 20.6% (8.7–37.9%), 55.9% (37.9–72.8%), and 82.4% (65.5–93.2%) for the same cutoffs, respectively. It may only be assumed that AUROC values, being usually quite high with such sensitivity and specificity values, could be close to the mean value of sensitivity and specificity, likely around 0.59, 0.71, and 0.66 for 20 kPa SSM, 40 kPa SSM, and 70 kPa SSM, respectively. Failure to provide the optimal cutoff explicitly makes it impossible to relate their results to the results of any other research.

Zhang F. et al. used two-dimensional SWE (2D-SWE) for HREVs prediction in patients with cirrhosis, obtaining an AUROC value of 0.868 (95% CI: 0.817–0.918) in the training cohort (TC) [47]. They did not report the value from the validation cohort. In our opinion, results from training cohorts should not be included in classic systematic reviews, but only where there are sufficiently powerful statistical tools to discriminate biased TC results.

In multiple studies, the authors were close to obtaining enough data to determine the AUROC as they were looking for an optimal cutoff. Note that AUROC is reported for multiple values when sensitivity and specificity are calculated for a single value. Similarly, in many cases, the authors could possibly calculate the ICC but did not. Some of the search results were not included because the SSM was not provided, even though it would have added value to the discussion of the results obtained by the authors. For instance, Cheng et al., 2024 [48], although assessing liver stiffness measurement (LSM) and spleen size, did not evaluate SSM.

Forty-seven studies involving a total of 7995 patients were included (Table A1 in the Appendix A).

Despite very intensive research, especially on EVs, the quality of scientific evidence may still raise some methodological concerns (Table 1).

In most cases, the risk of bias was low across all domains. Often, however, not all the data necessary for the assessment were provided in the article or the abstract. In such cases, we were forced to assess the domain as unclear, even if we guessed that the issue was minor.

The most common issue that affected most of the articles assessed using the QUADAS-2 tool was the lack of mention of operator blinding. Most authors did not provide any information on this topic. Some, however, while they mentioned the blinding of the US operator, did not mention the blinding of the endoscopist.

Both the operator measuring the dependent variable and the operator measuring the reference standard should be blinded to each other’s results, and this should be indicated explicitly. Furthermore, blinding of operators to all clinical data was rarely assured, and if so, only to the endoscopy result.

Objections regarding the choice of reference standard were rare. In some cases, e.g., Lantinga et al. [49], we had some doubts about the reference standard used. Clinically significant portal hypertension (CSPH) was defined using clinical and imaging criteria based on the Baveno VII consensus. The hepatic venous pressure gradient (HVPG) was not used due to ethical concerns in patients without advanced disease. Although CSPH was based on recognized surrogate markers, the absence of the HVPG as a definitive gold standard limits the accuracy of classification.

In most cases, the lower scientific quality of the articles was due to the very idea, namely that the authors were looking for the effectiveness of detecting varicose veins, not portal hypertension, which would raise fewer doubts.

Study selection and data extraction did not identify clear evidence of publication bias. However, the small number of studies on MNs and the exclusion of untranslatable articles in languages other than English may still contribute to unrecognized bias.

Given the focused scope and limited number of eligible studies, structured forms were not deemed necessary. Instead, consistency was maintained through parallel extractions and mutual verification, which effectively mitigated the risk of data discrepancies.

Assessment of methodological quality using the QUADAS-2 tool (Table 1) revealed that the overall risk of bias was low in most studies, particularly in the domains related to patient selection (D1) and flow and time (D4), where we found no obvious errors or possible sources of bias for most studies. A larger percentage of studies, however, demonstrated unclear or high risk of bias in the domains of the index test (D2) and reference standard (D3). This issue is therefore more relevant to the presented data. The main reasons for this were the lack of information about whether the operator performing the index test was blinded to the reference standard and vice versa, and the use of suboptimal or surrogate reference standards. It should be noted that the main reason for the negative bias assessment was the omission of information about the US operator’s blindness to the reference standard, even though the context and general description of the studies indicated that the operator was likely blinded in all cases. When double-blinding was not available, we sought definitive information defining the time sequence, as the operator is undoubtedly blinded to the reference results, which are not yet available. Even such a definitive definition was not available in almost all studies addressed by the comments shown in the table.

In the context of studies assessing splenic stiffness in the diagnosis of portal hypertension or varices, the gold standard of reference would be direct measurement of the pressure gradient in the hepatic veins, but this is an invasive technique and therefore could not be expected from all authors. As a result, many studies relied on clinical or endoscopic criteria (e.g., the presence of esophageal varices or the Baveno consensus definition) as the reference standards.

Sufficient operator blinding in these studies would require that the person performing the elastography examination be unaware of the patient’s clinical condition or diagnostic result, as defined by the reference standard (e.g., endoscopy or HVPG), and the person assessing the reference standard be unaware of the elastographic results. Furthermore, blinding broader clinical data, such as laboratory or imaging test results, would reduce the risk of incorporation bias. However, it should be noted that this is not entirely possible for the sonographer, who must consider the patient’s physical characteristics to conceptualize the precise method of measurement, thus having some understanding of the risk of a given outcome. It is even more unrealistic to expect complete blinding of the endoscopist to clinical data, given the risk of complications, which must be mitigated by conducting the probe and assessing whether there are contraindications to the examination.

Considering the combined impact of the index test domains and reference standards, as well as the presence or absence of appropriate blinding and valid gold standards, only approximately 12–15 of the 47 included studies can be considered demonstrably methodologically sound. These studies were characterized by a low risk of bias in all four domains. On the other hand, approximately 10 studies were (as shown in Table 1) at a high risk of bias in at least one domain or lacked essential methodological safeguards, such as operator blinding or the use of a valid reference standard, which made the obtained results less reliable.

**Table 1:** QUADAS-2 evaluation results.

Study	Risk of Bias by Domain				Applicability by Domain		
	D1	D2	D3	D4	D1	D2	D3
[50]	Low	Unclear	Unclear	Low	Low	Low	Low
[51]	High	Unclear	Unclear	High	Low	Low	Low
[52]	Unclear	Unclear	High	Unclear	Low	Low	Low
[53]	Low	Low	Low	Low	Low	Low	Low
[54]	Low	Low	Unclear	Low	Low	Low	Low
[55]	High	High	Unclear	Low	Low	Low	Low
[56]	Low	Low	Unclear	Low	Low	Low	Low
[57]	Low	Low	Unclear	Low	Low	Low	Low
[58]	Low	Unclear	High	Low	Low	Low	Low
[59]	Low	Low	Unclear	Low	Low	Low	Low
[60]	Low	Low	Unclear	Low	Low	Low	Low
[61]	Low	Unclear	Low	Low	Low	Low	Low
[62]	Low	Low	Unclear	Low	Low	Low	Low
[63]	Low	Low	Low	Low	Low	Low	Low
[64]	Low	Low	Unclear	Low	Low	Low	Low
[65]	Low	Low	Unclear	Low	Low	Low	Low
[66]	Low	Low	Low	Low	Low	Low	Low
[67]	Low	Low	Low	Low	Low	Low	Low
[68]	Low	Low	Low	Low	Low	Low	Low
[69]	Low	Low	Low	Low	Low	Low	Low
[70]	Low	Low	Unclear	Low	Low	Low	Low
[71]	Low	Unclear	Unclear	Low	Unclear	Low	Low
[72]	Low	Low	Low	Low	Low	Low	Low
[49]	Low	Low	Low	Low	Low	Low	Low
[73]	Low	Low	Unclear	Low	Low	Low	Low
[74]	Low	Low	Unclear	Low	Low	Low	Low
[75]	Low	Low	Unclear	Low	Unclear	Low	Low
[76]	Low	Low	Low	Low	Low	Low	Low
[77]	Low	Low	Unclear	Low	Low	Low	Low
[78]	Low	Low	Low	Low	Low	Low	Low
[79]	Low	Low	Unclear	Low	Low	Low	Low
[80]	Low	Unclear	Unclear	Low	Low	Low	Low
[81]	Low	Unclear	High	Low	Low	Low	Low
[82]	Low	Unclear	Unclear	Low	Low	Low	Low
[83]	Low	Low	Low	Low	Low	Low	Low
[84]	Low	Unclear	Low	Low	Low	Low	Low
[85]	Low	Low	Unclear	Low	Low	Low	Low
[86]	High	Unclear	Unclear	Low	Low	Low	Low
[87]	Low	Unclear	Low	Low	Low	Low	Low
[88]	High	Low	Low	Low	Low	Low	Low
[89]	Low	Low	Unclear	Low	Low	Low	Low
[90]	Low	Unclear	Unclear	Low	Low	Low	Low
[91]	Low	Low	Low	Low	Low	Low	Low
[92]	Low	Unclear	Unclear	Low	Low	Low	Low

Publication bias was assessed qualitatively by analyzing the availability and completeness of reported results in included studies. The search strategy aimed to minimize bias by including studies regardless of the results and referencing relevant excluded studies.

### 3.1. Results for SSM in MNs

The following five studies directly addressed the discriminatory power of US elastographic methods for examining the spleen in hematological applications [50,51,52,53,91]. These studies are described in Table A2 in the Appendix A.

Benedetti et al. obtained SSM values associated with worse progression-free survival (PFS) in patients with myelofibrosis, with SS above 40 kPa significantly linked to poor outcomes (HR = 3.2). SS correlated with the extent of bone marrow fibrosis and was higher in advanced fibrotic stages MF-2, MF-3 compared to pre-fibrotic/early fibrotic stages MF-0, MF-1. PFS rates were 63% in advanced stages versus 85% in early stages [50].

Sansone et al. obtained SSM in MF patients significantly higher than in healthy volunteers (pSWE 40.9 vs. 26.3 kPa, *p* < 0.001; 2D-SWE 34.9 vs. 20.1 kPa, *p* < 0.001) [51].

Ekinci et al. [52] measured splenic stiffness by shear wave elastography and reported median values of 0.82 m/s in healthy volunteers (HVs), 1.41 m/s in patients with PV/ETH, and 2.32 m/s in those with secondary myelofibrosis (SMF). Spleen stiffness correlated strongly with the histological grade of bone-marrow fibrosis (Spearman r = 0.757).

Rigamonti et al. obtained median SSM values in Group A with chronic liver disease (CLD) at 26.5 kPa, Group B (myeloproliferative neoplasms) 26.3 kPa, and Group C (healthy) 16.1 kPa; interobserver agreement ICC 0.90 [53].

Rigamonti et al. calculated median SSM values in the CLD group at 26.5 kPa (AUROC = 0.76, 95% CI 0.65–0.87), in the MNs Group B at 26.3 kPa, and in healthy patients at 16.1 kPa. Interobserver agreement ICC ranged from 0.84 to 0.92, slightly better in patients with splenomegaly and cirrhosis [53].

Yalçın et al. obtained median shear wave velocity (SWV) significantly higher in the hepatoportal (3.85 m/s) and myeloproliferative (3.42 m/s) groups compared to the infectious (2.66 m/s) and control (2.22 m/s) groups. Cutoff values and their sensitivities and specificities were 3.42 m/s (sensitivity 80.9%, specificity 56.5%) for distinguishing hepatoportal from myeloproliferative etiology; 3.02 m/s (sensitivity 100%, specificity 100%) for distinguishing hepatoportal from infectious etiology; and 2.84 m/s (sensitivity 91.3%, specificity 88.2%) for distinguishing myeloproliferative from infectious etiology [91].

For the synthesis in the part regarding the possibilities and limitations of the use of SSM in diagnosing MNs (Table A3 in the Appendix A). The overall risk of bias is low, considering the individual bias assessment of all sources. The evidential value in this regard is, however, low due to the critically small number of studies (only five papers).

### 3.2. Results for SSM in Other Applications

In total, 42 studies addressed the gastroenterological and hematological applications of spleen US elastography, mainly in the field of esophageal varices (Table A3 in the Appendix A). Table 2 provides the US methods used in the included studies.

In the case of the discriminatory power of individual US methods, the risk of bias is generally low across individual studies, and the evidential power is relatively high because the number of studies conducted is relatively large (Table A3 in the Appendix A).

### 3.3. Reproductivity of US SSM

Inter-operator agreement has been examined in significantly fewer studies than discriminatory power (Table 3). The main measure of repeatability was the ICC.

With respect to the ICC, the bias is again rather low, but the number of studies is small.

## 4. Discussion

The body of evidence concerning the use of SSM in MNs is notably limited. Only four original studies [50,51,52,53] were included, with a combined total of fewer than 300 MNs patients overall. The studies varied considerably in design, including both retrospective [50,53] and prospective [51,52] approaches, introducing potential heterogeneity in patient selection and data quality. The only positive aspect here is the fact that the studies covered a relatively wide group of MNs, suggesting that elastographic methods of spleen diagnostics can indeed serve as a marker of pathological myeloproliferation. However, the sample sizes within individual MN studies were also relatively small, and external validation cohorts were generally absent. The risk of bias in the MN cohorts, remains elevated due to limited patient numbers.

While the body of evidence regarding spleen elastography in non-MN conditions such as CLD, cirrhosis, PH, and EV is obviously larger and more diverse, it also presents certain important limitations. While some very large studies were included [67,84,85], others had relatively small cohorts [58,60,73,78]. Most studies were prospective [49,55,56,57,61,62,63,72,73,74,75,76,77,79,81,82,83,84,85,87,88,91,93,94], but some were retrospective [56,59,67,82,83,86,91], cross-sectional [49,58,60,78,79,85], or quasi-experimental [55], causing the variability in methodology. A key limitation is probably the heterogeneity of disease severity among included patients, as some studies focused on early-stage cirrhosis [62,63], while others included patients with clinically significant PH or high-risk EVs [70,72,77], and therefore generally in a worse functional condition of the digestive system and liver. This variation affects the comparability of results and generalizability across different clinical settings.

The obtained studies can be divided into two groups based on disease type: MNs and others, where conditions associated with specific hepatic portal circulation are present, ranging from associated risks to actual pathologies, including those affecting the esophagus.

Four elastographic techniques were used for MNs, of which ARFI was used in two studies—one of which achieved an “excellent” result (AUROC ≥ 0.9) and the other “moderate” (AUROC 0.7–0.8). 2D-SWE was used in three studies, one of which achieved a “moderate” result and two “poor” results (AUROC < 0.7). Similarly, p-SWE was evaluated in three studies: one had a “moderate” result and two had “poor” results. 2D-SWE-STE was used in one study and achieved a “good” result (AUROC 0.8–0.9). A total of nine studies in the MNs group provided only isolated evidence of high diagnostic performance, with the majority of results falling below the “good” level.

For non-myeloproliferative diseases, the number of data was significantly greater, and the distribution of results was clearly more favorable. ARFI was used in eight studies, four of which achieved “excellent” results and four “good” results. 2D-SWE appeared in twelve studies, two of which achieved “excellent” results and ten “good” results. Other techniques, such as impulse elastography (two studies), TE (four studies), VCTE (four studies), p-SWE (three studies), SSPI by pSWE, 2D-SWE-STE, EUS-elastography, and VTQ (one study each), also achieved high performance, most exceeding the “good” threshold. No “poor” results were reported in this group.

Against the above background, it seems easy to compare the state of research on the use of spleen elastography results as a marker or predictor of systemic pathologies. Spleen elastography has been widely studied in conditions other than MNs, particularly in cohorts with CLD, cirrhosis, HP, and EVs. In these settings, elastography techniques such as acoustic radiation force impulse (ARFI) have proven highly effective with AUROC values often exceeding 0.90. For example, ARFI demonstrated an AUROC of 0.996 for predicting small esophageal varices in patients with hepatitis B-related cirrhosis [84], while 2D-SWE achieved an AUROC of 0.98 for detecting HRSVs in idiopathic portal hypertension [88]. Similarly high performances were observed across multiple studies focused on variceal prediction in cirrhosis, such as ARFI showing an AUROC of 0.969 in CHC/CHB for large EVs [76], ARFI with an AUROC of 0.881 for HREV in CHB cirrhosis [84], and 2D-SWE reaching an AUROC of 0.96 for HREV in CHB [88]. Additional studies confirmed excellent results, including SSPI by pSWE for compensated cirrhosis (AUROC 0.95) [65], VCTE at 100 Hz in MAFLD for CSPH (AUROC 0.95) [82], and impulse elastography (FibroScan Echosens) with an AUROC of 0.906 for EV prediction in CLD [81].

Additionally, other techniques such as VCTE [53,68,71,82,83], SSM at 100 Hz [94], and impulse elastography [81] have shown acceptable to excellent performance in the diagnosis of CSPH, EVs, and high-risk varices. In CLD cohorts, 2D-SWE-STE [61], VCTE [83], and 2D-SWE [61,81] achieved generally acceptable to good performances. In diagnosing CSPH, 2D-SWE [81,90,92], TE by FibroScan [92], and VCTE [68] performed well, with VCTE at 100 Hz demonstrating excellent AUROC in patients with MAFLD [82]. In the detection of EVs, techniques like SSM@100 Hz [94], VCTE [53,71,83], 2D-SWE [56], TE by FibroScan [78], ARFI [59,76,84], and VCTE at 100 Hz [71] all showed acceptable to good AUROC values, while impulse elastography [81] and SSPI by pSWE [65] reached excellent AUROC values. For large EVs, ARFI [76,84] and impulse elastography [81] offered good to excellent discriminatory power. In diagnosing high-risk varices, ARFI [77,84], 2D-SWE [62,63,72], SSI [57], SSM@100 Hz [94], TE [78,79,80], and VCTE [71,83] performed acceptably to well, while p-SWE [63], TE by FibroScan [64], and new fusion devices [79] achieved excellent results. PH was studied less often but showed acceptable to good diagnostic performance with ARFI [69], p-SWE [54], EUS-elastography [95], and TE [58].

When it comes to MNs, the current research remains limited, yet the available studies consistently highlight the potential value of SSM in this setting. Several elastographic techniques, including ARFI, p-SWE, and 2D-SWE, have been evaluated so far. SSM, whether based on spleen stiffness alone or shear wave velocity (SWV), appears to offer a valuable, non-invasive method for both diagnosing and monitoring myeloproliferative neoplasms. These measurements demonstrate the ability to differentiate MNs from other conditions, assess disease severity, and provide prognostic data.

Higher spleen stiffness values have been shown to correlate strongly with the degree of bone marrow fibrosis and adverse clinical outcomes. In particular, SSM values exceeding 40 kPa were associated with significantly worse progression-free survival, with a hazard ratio of 3.2 for poor outcomes, and lower survival rates in advanced fibrotic stages compared to early stages [50]. Furthermore, studies demonstrated that patients with MF presented significantly higher spleen stiffness values compared to healthy volunteers, using both p-SWE and 2D-SWE modalities [51]. Another important finding is the strong correlation between spleen stiffness and the histological grade of bone marrow fibrosis, with a Spearman coefficient of 0.757 [52]. This may suggest that SSM could potentially serve not only as a diagnostic marker but also as a tool for risk stratification and prognosis in MNs.

Among the elastographic techniques evaluated, ARFI appears particularly promising. Although based on a limited number of patients, ARFI showed excellent performance in distinguishing myeloproliferative from infectious splenomegaly, achieving an AUROC of 0.963 [91]. Furthermore, it perfectly differentiated hepatoportal from infectious causes (AUROC 1.000) [91], although the differentiation between hepatoportal and myeloproliferative splenomegaly was less precise (AUROC 0.776) [91]. Therefore, if proven by further research, ARFI may be especially useful for non-invasive etiological assessment of splenomegaly in MN patients, although a validation is needed.

2D-SWE was found to detect the presence of MF with acceptable accuracy (AUROC 0.752) but showed poor discrimination between fibrosis grades in PV and ET [51]. Similarly, p-SWE acceptably distinguished low- versus high-grade fibrosis (AUROC 0.702) and detected myelofibrosis (AUROC 0.723), but again failed to reliably separate fibrosis stages in PV and ET [51]. These observations underline a persistent challenge in using elastography for precise fibrosis staging in MNs outside of overt myelofibrosis.

Beyond MN-specific studies, comparative data on spleen stiffness from broader patient cohorts also reinforce the relevance of SSM. In one study, patients with CLD and MNs had comparable median spleen stiffness values (26.5 kPa and 26.3 kPa, respectively), both markedly higher than those of healthy controls (16.1 kPa), with excellent interobserver agreement (ICC 0.84–0.92) [53]. Moreover, SWV measurements were significantly higher in hepatoportal and myeloproliferative groups compared to infectious and control groups, further supporting the potential of SSM to distinguish MNs based on spleen biomechanical properties [91].

Measurement reproducibility appears satisfactory in all clinical settings despite the fact that measuring the spleen is particularly difficult compared to examining the much larger liver. In the studies focused on hepatological conditions, ICCs were reported as good to excellent for VCTE, p-SWE, and 2D-SWE-STE [53,66,84], similarly as in Philadelphia-negative MNs [53]. Importantly, while spleen size affected measurement ease, good to excellent reproducibility was preserved even in patients without splenomegaly [84,94]. Nevertheless, the presented data suggest that the size of the spleen is important for obtaining appropriate results. The small number of studies that prove its usefulness also in patients without splenomegaly does not allow for a clear determination of whether the objectivity of the test will be sufficiently high for screening purposes, and not only for determining the etiology of splenomegaly.

The overall risk of bias across all included studies supports the strength of the evidence, particularly regarding diagnostic performance in conditions other than MN. However, in MN-related studies, the limited number and smaller sample sizes reduce certainty, despite the predominantly low estimates of bias.

The current research achievements on spleen stiffness measurement in MNs allow us to identify the most promising techniques for further study. This includes 2D-SWE, ARFI, EUS-elastography, impulse elastography (FibroScan Echosens), p-SWE, SSPI by pSWE, TE (by FibroScan or new fusion devices), and VCTE at 100 Hz. However, it must be noted that the greater the degree of portal hypertension or the severity of esophageal varices, the better the discriminatory power of these methods, although in some studies, smaller varices were also detected effectively.

Despite the growing interest in SSM, technical limitations remain a significant problem, particularly in patients without splenomegaly. Unlike the liver, the anatomical location of the spleen complicates both image acquisition and elastographic signal stability, especially when high-frequency transducers are required for resolution, but their penetration depth is limited [40,41]. These problems are exacerbated in patients without splenomegaly, where the acoustic window is more limited and the target tissue is narrower. In such cases, the risk of bias arises. This is a result of operators being forced to place regions of interest in suboptimal parenchymal zones, which increases the risk of including various structures that will adversely modulate the measurement result. The measurement result will no longer be a function of a single variable, such as splenic stiffness, but instead become a function of multiple variables. Examples include vascular structures, hilar tissue, and edge artifacts. Their stiffness will undesirably modulate the obtained measurement result.

Another issue is the abnormally lower baseline stiffness of the non-enlarged spleen, typically in the range of 2.2–2.6 m/s or 15–20 kPa, depending on the technique and reference values [52,53,91]. This range narrows the difference between normal and abnormal values, making it difficult to detect subtle pathological changes and increasing the risk of their superimposition on normal variation. Such subtle distinctions are particularly problematic in the early stages of MN or in post-treatment monitoring, where splenic stiffness may regress to baseline values. Although some studies, such as Rigamonti et al., demonstrate high interobserver agreement in MNs using VCTE (ICC = 0.90; 0.83–0.94) [53], these data are primarily derived from cohorts with overt splenomegaly or cirrhosis. In patients without splenomegaly, however, reliable measurement requires a high level of operator expertise and frequent repeat acquisitions. While 2D-SWE and ARFI-based p-SWE offer significant simplification in localizing the region of interest and may perform better within such limitations, reproducibility in small, normal spleens remains largely anecdotal, i.e., cannot be considered robust given the number of studies reporting ICC. These limitations question the feasibility of using SSM as a routine screening or monitoring tool in early or benign spleens and suggest the need for more rigorous validation studies, including stratification by spleen size and standardized acquisition quality criteria. However, it should be noted that the ICC and AUROC values presented in the studies are a strong argument for recognizing that the evolution of elastographic techniques, especially in the form of new modalities 2D-SWE and p-SWE based on ARFI, gives grounds to assume that the possibility of imaging in the desired area of interest will improve enough that the size of the non-enlarged organ will not be a problem in the perspective of further studies.

At this stage, spleen elastography, particularly ARFI and 2D-SWE, can already be considered prospective non-invasive tools for distinguishing between different causes of splenomegaly in MNs. At this stage, spleen elastography, particularly ARFI and 2D-SWE, shows promise as a non-invasive tool to help differentiate splenomegaly caused by MNs from that due to other etiologies, such as infectious or congestive causes. Although not sufficient to classify MN subtypes, increased stiffness values measured by these techniques may support the suspicion of a myeloproliferative origin in patients with unexplained splenomegaly.

However, as mentioned above, several aspects still require further investigation. There is a clear need to standardize spleen stiffness cutoff values specific to MNs and to validate elastographic techniques in broader MN populations, including patients without splenomegaly. Moreover, newer techniques that have shown excellent diagnostic accuracy in CLD could represent promising future directions for MN research.

Significant challenges still need to be addressed. The inherent heterogeneity of MNs, encompassing diseases such as myelofibrosis, polycythemia vera, and essential thrombocythemia, complicates the establishment of unified diagnostic standards. Technical difficulties, including measurement depth and patient-related factors such as spleen size or body habitus, can impact reproducibility and accuracy. Additionally, the differentiation of subtle fibrosis grades, particularly in the absence of splenomegaly, remains a difficult goal yet to be achieved.

To move forward, larger multicenter studies are needed, particularly those involving patients without splenomegaly, using standardized elastography techniques across different operators and devices. Integration of SSM into clinical practice for MNs would also require cross-validation with other diagnostic tools.

## 5. Conclusions

Myeloproliferative diseases and bone marrow fibrosis affect the biophysical characteristics of the spleen in a similar, although not the same way, as the phenomenon of portal hypertension. Diagnostic methods including the use of ARFI, p-SWE, TE, and 2D-SWE within complex algorithms can aid in their diagnosis and monitoring. Their application, however, requires a number of new studies, the planning of which may be aided by the achievements of the use of SSM in the diagnosis of HP and EVs.

In studies involving MNs, ARFI demonstrated the highest performance (AUROC values close to one), while 2D-SWE and p-SWE achieved moderate to poor results, indicating their limited usefulness in precisely differentiating fibrosis stages at this stage.

In liver disease, ARFI and 2D-SWE consistently achieved high AUROC values (often >0.9), demonstrating greater reproducibility and robustness than p-SWE and TE, which still yielded good but more variable results.

In new studies on MN patients, it is important to measure inter-operator repeatability and agreement, especially in patients without splenomegaly, because spleen measurement is difficult and may still raise doubts about its validity.

## Figures and Tables

**Table 2:** Methods used in the studies.

Elastographic Method (Equipment/Approach)	No. of Sources	Reference IDs
Transient elastography		
(FibroScan^®^, Echosens, Paris, France, VCTE, 50 Hz or 100 Hz spleen probe)	7	[29,35,39,44,53,61,68]
Point shear wave elastography (pSWE), including ARFI-based single-ROI measurements	9	[25,26,30,31,34,37,38,40,65]
Two-dimensional shear wave elastography (2D-SWE)		
(incl. SuperSonic Imagine SSI^®^, STE, etc., Aix-en-Provence, France)	6	[28,32,33,34,66,68]
Endoscopic ultrasound strain elastography	1	[52]

TE—transient elastography; VCTE—vibration-controlled transient elastography (FibroScan^®^); pSWE—point shear wave elastography; ARFI—acoustic radiation-force impulse (technology underpinning pSWE); ROI—region of interest (single measurement window in pSWE); 2D-SWE—two-dimensional shear wave elastography; SSI—SuperSonic Imagine^®^—commercial platform implementing 2D-SWE; STE—supersonic transient elastography (trade name for SSI 2D-SWE mode).

**Table 3:** ICCs presented in each study.

[53]	VCTE	CLD	Overall	ICC: 0.90 (0.88–0.92)
[53]	VCTE	CLD	CLD with splenomegaly	0.91
[53]	VCTE	CLD	CLD without splenomegaly	ICC: 0.87
[53]	VCTE	CLD	CLD with cirrhosis	ICC: 0.90
[53]	VCTE	CLD	CLD without cirrhosis	ICC: 0.84
[53]	VCTE	Philadelphia-negative MNs	Overall	ICC: 0.90 (0.83–0.94)
[85]	VCTE	CHB cirrhosis	50 Hz vs. 100 Hz	ICC: 0.66 (95% CI, 0.58–0.73)
[66]	p-SWE	CLD	TIPS dysfunction	ICC: 0.90 (95% CI, 0.81–0.94)
[84]	2D-SWE-STE	CHB	Overall	ICC: 0.964
[84]	2D-SWE-STE	CHB	CHB without splenomegaly	ICC: 0.980
[84]	2D-SWE-STE	CHB	CHB with splenomegaly	ICC: 0.940

VCTE—vibration-controlled transient elastography (FibroScan^®^); pSWE—point shear wave elastography; ARFI—acoustic radiation-force impulse (technology underpinning pSWE); 2D-SWE—two-dimensional shear wave elastography; CLD—Chronic Liver Disease, MNs—Myeloproliferative Neoplasms, CHB—Chronic hepatitis B, TIPS—transjugular intrahepatic portosystemic shunt, ICC—intraclass correlation.

## Data Availability

Not applicable.

## Abbreviations

The following abbreviations are used in this manuscript:

2D-SWE	Two-Dimensional Shear Wave Elastography
ARFI	Acoustic Radiation Force Impulse
AUC	Area Under Curve
AUROC	Area Under the Receiver Operating Characteristic Curve
CML	Chronic Myeloid Leukemia
CSPH	Clinically Significant Portal Hypertension
ETH	Essential Thrombocythemia
EUS	Endoscopic Ultrasound
EUS-elastography	Endoscopic Ultrasound Elastography
EVs	Esophageal Varices
HREVs	High-Risk Esophageal Varices
HVPG	Hepatic Venous Pressure Gradient
ICC	Intraclass Correlation Coefficient
LSM	Liver Stiffness Measurement
MNs	Myeloproliferative Neoplasms
MYF	Primary myelofibrosis
PRISMA	Preferred Reporting Items for Systematic Reviews and Meta-Analyses
p-SWE	Point Shear Wave Elastography
PV	Polycythemia vera
QUADAS-2	Quality Assessment of Diagnostic Accuracy Studies 2
SSI	Supersonic Shear Imaging
SSM	Spleen Stiffness Measurement
SWE	Shear Wave Elastography
SWV	Shear Wave Velocity
TC	Training Cohort
TE	Transient Elastography
US	Ultrasound
VCTE	Vibration-Controlled Transient Elastography

## Appendix A

**Table A1:** Studies included in the review.

Study	Type of the Study	Number of Patients	Medical Conditions (Research Group)
MNs			
Benedetti et al. [50]	Retrospective	70	Myelofibrosis and other Philadelphia-negative MNs
Sansone et al. [51]	Prospective	218 (143 with MNs)	Myeloproliferative neoplasms vs. healthy volunteers
Ekinci et al. [52]	Prospective	121 (69 with MNs)	Polycythemia vera, essential thrombocythemia, and secondary myelofibrosis
Rigamonti et al. [53]	Retrospective	420 (63 with MNs)	Reproducibility of spleen stiffness measurement in Philadelphia-negative MNs and CLD
**Other conditions**			
Ahmad et al. [54]	Prospective	50	Portal hypertension in patients with liver disease of various etiologies
Arya et al. [44]	Quasi-experimental	100	CLD and prediction of EVs
Cho et al., 2022 [56]	Retrospective	65	HBV-related cirrhosis and prediction of EVs
Cho et al., 2022 [57]	Retrospective	274	cACLD (comparing non-invasive criteria for endoscopy avoidance)
Dajti et al. [58]	Cross-sectional	34 (with valid SSM)	Pediatric and adult CF patients
Darweesh et al. [59]	Cross-sectional	90	HCV patients for EVs prediction
Dogan et al. [60]	Case–control	100 (40 brucellosis, 60 control)	Acute brucellosis and healthy controls
Dong et al. [61]	Prospective	100	CHB
Fofiu et al., 2021 [62]	Prospective	132	Compensated cirrhosis for HREV prediction
Fofiu et al., 2021 [63]	Prospective	107	Compensated cirrhosis (predicting varices needing treatment)
Gaspar et al. [64]	Prospective	209	Cirrhotic patients (prediction of high-risk varices)
Giuffrè et al. [65]	Cross-sectional	210	Cirrhosis (presence of EVs)
Giunta et al. [66]	Prospective	24	TIPS dysfunction (before and after TIPS placement/revision)
Huang et al. [67]	Retrospective	741 (407 with compensated cirrhosis)	Compensated cirrhosis (HBV and other etiologies) comparing ARFI-based and Baveno VI strategies
Jachs et al. [68]	Prospective	407	cACLD (CSPH prediction)
Jain et al. [69]	Cross-sectional	90	Cirrhosis (prediction of EVs)
Karagiannakis et al., 2022 [70]	Prospective	177	Cirrhosis (follow-up of clinical events over 31 months)
Kurniawan et al., 2024 [71]	Prospective	141	Cirrhosis (diagnostic accuracy of SSM, LSM, APRI for EVs)
Kuroda et al. [72]	Prospective	111	Cirrhosis (HV-PV gradient, spleen stiffness, and liver stiffness for high-risk EV)
Lantinga et al., 2023 [49]	Prospective	118	Portal hypertension prediction by liver VCTE
Muñoz-Codoceo et al. [73]	Cross-sectional	97	HCV-related cirrhosis post-antiviral therapy (gastroesophageal varices)
Nardelli et al. [93]	Cross-sectional	51	Hepatosplenic schistosomiasis (EVs)
Mnif et al. [74]	Retrospective	84	CHB (prediction of EVs)
Odriozola et al. [75]	Retrospective	85	Mainly MAFLD (diagnostic performance of SSM for clinically significant portal hypertension)
Peagu et al. [76]	Prospective	175	HBV/HCV-related cirrhosis (diagnosing EVs)
Petrisor et al. [77]	Prospective	130	Cirrhosis stratified by bleeding risk
Robles-Medranda et al. [95]	Prospective	61	Cirrhosis (predicting portal hypertension using endoscopic ultrasonographic elastography)
Stefanescu et al. [94]	Prospective	260	Diagnosis of EVs using novel spleen-dedicated FibroScan^®^ (SSM@100 Hz) vs. standard methods
Takuma et al. [78]	Prospective	20	Gastric varices (pre/post-balloon-occluded retrograde transvenous obliteration)
Tanaka et al. [79]	Prospective	200	Cirrhosis (predicting EVs with a new TE+ultrasound fusion de-vice)
Vanderschueren et al. [80]	Retrospective	377	Ruling out varices needing treatment using TE SSM algorithms vs. Baveno VI criteria
Wang et al. [81]	Retrospective	107	Compensatory viral cirrhosis (portal vein pressure)
Williams et al. [82]	Prospective	60	MAFLD—diagnosis of cirrhosis and EVs
Xu X et al. [83]	Prospective	320	B-viral cirrhosis (predicting EV grades and high-risk EV)
Zhang W et al. [84]	Prospective	603	CHB—diagnosing cirrhosis across various ALT levels
Zhang X et al. [85]	Prospective	504	HBV-related cirrhosis (detecting high-risk varices using VCTE with AGD as reference)
Zhang Z et al. [86]	Cross-sectional	173	Validating Baveno VI criteria and spleen diameter (including SSM) to rule out high-risk varices
Zhou et al., 2021 [87]	Prospective	107	Gastroesophageal varices severity post-TIPS (2D-SWE measurement)
Zhou et al., 2023 [88]	Retrospective	188 (86 IPH, 102 HBV)	Idiopathic portal hypertension vs. HBV (presence of high-risk varices)
Zhu et al., 2020 [89]	Prospective	89	CLD in TIPS patients (SSM to predict survival)
Zhu et al., 2020 [90]	Retrospective	58	HBV-related cirrhosis (LSM and SSM correlation with HVPG)
Yalçın et al. [91]	Cross-sectional	61	Splenomegaly of various etiologies vs. healthy controls
Grgurevic et al. [92]	Prospective	76	Spleen stiffness vs. HVPG for diagnosing CSPH and HRV

MNs: myeloproliferative neoplasms; CLD: chronic liver disease; EVs: esophageal varices; HBV: hepatitis B virus; cACLD: compensated advanced chronic liver disease; CF: cystic fibrosis; HCV: hepatitis C virus; CHB: chronic hepatitis B; HREV: high-risk esophageal varices; TIPS: transjugular intrahepatic portosystemic shunt; ARFI: acoustic radiation force impulse; CSPH: clinically significant portal hypertension; SSM: spleen stiffness measurement; LSM: liver stiffness measurement; APRI: aspartate aminotransferase-to-platelet ratio index; HV-PV: hepatic venous–portal venous; VCTE: vibration-controlled transient elastography; MAFLD: metabolic-associated fatty liver disease; TE: transient elastography; ALT: alanine aminotransferase; AGD: assessment of gastric dilatation.

**Table A2:** Studies involving patients with MNs.

Study	Study Desing	Population	Technique Used	Diagnostic Performance	Main Conclusions
[50]	Prospective observational study	70 patients with MNs (43 MF, 17 PV, 10 ET) and 20 HCs	B-mode US and pSWE for SSM and LSM	SS significantly higher in MF and PV compared to ET and HCs (*p* < 0.0001). SS strongly correlated with bone marrow fibrosis (*p* < 0.0001), with SS > 40 kPa associated with worse progression-free survival (HR 1.939). LSM increased only in MF vs. HCs (*p* = 0.001).	SSM by pSWE is a reliable non-invasive marker of bone marrow fibrosis and progression risk in MF and PV
[51]	Prospective observational study	218 participants (143 MNs: 63 MF, 33 PV, 46 ET, and 75 HCs)	pSWE and 2D-SWE for LSM and SSM, combined with B-mode and Doppler US	SSM significantly higher in MF vs. ET, PV, and HCs (*p* < 0.001). ROC analysis for MF detection yielded AUROC 0.723 for pSWE (30.9 kPa) and 0.752 for 2D-SWE (25.6 kPa). SSM correlated strongly with bone marrow fibrosis grades (*p* < 0.001).	SWE effectively differentiates MF from other MNs and HCs, and SSM correlates with bone marrow fibrosis
[52]	Retrospective observational study	121 participants (52 HCs, 52 PV and/or ET, 17 secondary myelofibrosis)	SWE for SSM	Correlated to bone marrow fibrosis and spleen size. Median SSM 0.82 m/s (HV), 1.41 m/s (PV/ET), and 2.32 m/s (SMF), with significant differences across groups (*p* < 0.001). SSM strongly correlated with bone marrow fibrosis degree (r = 0.757, *p* < 0.001).	SWE can differentiate SMF from PV/ET and HCs
[53]	Prospective multicenter observational study	420 participants (297 patients with CLD, 63 Philadelphia-negative MNs, and 60 HCs)	VCTE with a spleen-dedicated module (SSM@100 Hz), alongside LSM	Median SSM values were 26.5 kPa (CLD), 26.3 kPa (MPNs), and 16.1 kPa (HCs). ICC = 0.90), with AUROC = 0.76 for detecting EVs in cirrhotic patients (cutoff ≥ 40 kPa, sensitivity 85%, specificity 54%).	SSM using the spleen-dedicated VCTE module is feasible, quick, and highly reproducible across different operators and patient groups
[91]	Prospective observational study	61 treatment-naïve patients with splenomegaly (21 hepatoportal, 23 MNs, 17 infectious) and 20 HCs	ARFI imaging, measuring SWV	Median SWV 3.85 m/s (hepatoportal), 3.42 m/s (myeloproliferative), 2.66 m/s (infectious), and 2.22 m/s (HCs). ROC cutoffs effectively distinguished etiologies: 3.42 m/s (hepatoportal vs. myeloproliferative; sensitivity 80.9%, specificity 56.5), 3.02 m/s (hepatoportal vs. infectious; sensitivity 100%, specificity 100), 2.84 m/s (myeloproliferative vs. infectious; sensitivity 91.3%, specificity 88.2).	ARFI-based SWE of the SSM is a useful, non-invasive tool to differentiate the etiology of splenomegaly

ARFI—Acoustic Radiation Force Impulse; AUROC—Area Under the Receiver Operating Characteristic Curve; CLD—Chronic Liver Disease; ET—Essential Thrombocythemia; EVs—Esophageal Varices; HCs—Healthy Controls; HR—Hazard Ratio; ICC—Intraclass Correlation Coefficient; kPa—kilopascal; LSM—Liver Stiffness Measurement; MF—Myelofibrosis; MNs—Myeloproliferative Neoplasms; pSWE—Point Shear Wave Elastography; PV—Polycythemia Vera; ROC—Receiver Operating Characteristic; SMF—Secondary Myelofibrosis; SS—Spleen Stiffness; SSM—Spleen Stiffness Measurement; SWE—Shear Wave Elastography; SWV—Shear Wave Velocity; US—Ultrasound; VCTE—Vibration-Controlled Transient Elastography.

**Table A3:** AUROC values presented in each study.

Study	Technique	Diseased	Condition Screened/Predicted	AUROC
MNs Discrimination				
[67]	ARFI	Splenomegaly	Myeloproliferative vs. infectious	0.963 (*p* < 0.001)
[67]	ARFI	Splenomegaly	Hepatoportal vs. myeloproliferative	0.776 (*p* < 0.001)
[51]	2D-SWE	MF	MF	0.752
[51]	p-SWE	Bone Marrow Fibrosis	Low vs. High-Grade Fibrosis	0.702
[51]	p-SWE	PV	Fibrosis Grade (0–1 vs. 2–3)	0.505
[51]	2D-SWE	PV	Fibrosis Grade (0–1 vs. 2–3)	0.479
[51]	2D-SWE	Essential Thrombocythemia (ET)	Fibrosis Grade (0–1 vs. 2–3)	0.412
[51]	p-SWE	ET	Fibrosis Grade (0–1 vs. 2–3)	0.389
[67]	ARFI	Splenomegaly	Hepatoportal vs. Infectious	1.000 (*p* < 0.001)
Other Conditions				
[84]	ARFI	CHB cirrhosis	Small EV	0.996 (95% CI 0.989–1.000)
[88]	2D-SWE	Idiopathic portal hypertension	HREV	0.98
[55]	ARFI	CLD	EV	0.98
[76]	ARFI	CHC/CHB	Large EV	0.969 (95% CI 0.935–0.99)
[88]	2D-SWE	CHB	HREV	0.96
[65]	SSPI by pSWE	Compensated cirrhosis	EV	0.95
[75]	VCTE at 100 Hz	MAFLD	CSPH	0.95
[64]	TE (FibroScan)	Cirrhosis	HREV	0.934 (95% CI 0.621–0.793)
[79]	TE—new fusion device	Cirrhosis	HREV	0.921
[74]	Impulse elastography (FibroScan Echosens)	CLD	EV	0.906 (*p* = 0.000)
[74]	Impulse elastography (FibroScan Echosens)	CLD	Large EV	0.906 (*p* = 0.000)
[60]	p-SWE	Acute brucellosis	Presence of acute brucellosis	0.903
[61]	2D-SWE-STE	CHB	Cirrhosis	0.90 (95% CI 0.81–1.00)
[63]	p-SWE	Compensated cirrhosis	HREV	0.90
[81]	2D-SWE	CHC/CHB	CSPH	0.895 (95% IC 0.834–0.957, *p* < 0.05)
[71]	VCTE at 100 Hz	Cirrhosis	EV	0.892 (95% CI 0.814–0.969, *p* < 0.0001)
[84]	ARFI	CHB cirrhosis	HREV	0.881 (95% CI 0.825–0.937)
[80]	TE	Advanced CLD	HREV	0.88
[92]	2D-SWE	Compensated advanced CLD	CSPH	0.877 (95% CI 0.792–0.963)
[76]	ARFI	CHC/CHB	EV	0.872 (95% CI 0.799–0.944)
[77]	ARFI	Cirrhosis	HREV	0.863 (95% CI 0.791–0.917, *p* < 0.001)
[66]	p-SWE	CLD	TIPS dysfunction	0.86 (95% CI 0.70–0.96)
[62]	2D-SWE	Compensated cirrhosis	HREV	0.86
[78]	TE	Cirrhosis	HREV	0.858
[92]	TE (FibroScan)	Compensated advanced CLD	CSPH	0.857 (95% CI 0.763–0.951)
[93]	TE (FibroScan)	Hepatosplenic schistosomiasis	EV	0.856 (95% CI 0.752–0.961, *p* = 0.001)
[90]	2D-SWE	CHB cirrhosis	CSPH	0.85 (95% CI 0.59–0.96)
[58]	TE (FibroScan)	Compensated CLD	PH	0.841 (95% CI 0.706–0.976)
[63]	2D-SWE	Compensated cirrhosis	HREV	0.84
[84]	ARFI	CHB cirrhosis	Large EV	0.838 (95% CI 0.738–0.938)
[86]	2D-SWE	Compensated advanced CLD	HREV	0.834
[61]	2D-SWE-STE	CHB	S > 3 liver fibrosis	0.83 (95% CI 0.73–0.92)
[81]	2D-SWE	CHC/CHB	Cirrhosis	0.827 (95% IC 0.770–0.884, *p* < 0.05)
[82]	VCTE	MAFLD	HREV	0.82 (95% CI 0.75–0.98, *p* < 0.001)
[72]	2D-SWE	Cirrhosis	HREV	0.82 (95% CI 0.75–0.90)
[78]	VTQ	Gastric varices undergoing ballooning	Exacerbation of EV	0.818
[95]	EUS-elastography	CLD	PH	0.815 (95% CI 70.7–92.2)
[90]	2D-SWE	CHB cirrhosis	Severe portal hypertension	0.81 (95% CI 0.55–0.97
[68]	VCTE	Compensated advanced CLD	CSPH	0.804 (95% CI 0.743–0.864]
[70]	2D-SWE	Cirrhosis	1-year death or liver transplantation in high-risk patients	0.80 (*p* < 0.001)
[92]	TE (FibroScan)	Compensated advanced CLD	HREV	0.797 (95% CI 0.682–0.912)
[92]	2D-SWE	Compensated advanced CLD	HREV	0.795 (95% CI 0.687–0.904)
[94]	SSM@100 Hz	CLD	EV	0.782
[94]	SSM@100 Hz	CLD	HREV	0.780
[82]	VCTE	MAFLD	Cirrhosis	0.78 (95% CI 0.70–0.85, *p* < 0.001)
[56]	2D-SWE	CHB cirrhosis	HREV	0.778 (95% CI 0.658–0.872)
[89]	p-SWE	CLD	Survival with TIPS	0.769 (95% CI 0.657–0.881)
[56]	2D-SWE	CHB cirrhosis	EV	0.767 (95% CI 0.646–0.863)
[59]	ARFI	CHC advanced liver fibrosis	EV	0.76 (*p* = 0.000)
[53]	VCTE	CLD	EV	0.76 (95% CI 0.65–0.87)
[57]	SSI	Compensated advanced CLD	HREV	0.757
[94]	SSM@100 Hz	CLD	HREV	0.756
[82]	VCTE	MAFLD	EV	0.74 (95% CI 0.61–0.84, *p* < 0.001)
[87]	2D-SWE	Patients after TIPS	Severe gastroesophageal varices	0.739
[94]	SSM@100 Hz	CLD	EV	0.728
[77]	p-SWE	MF	MF	0.723
[94]	SSM@50 Hz	CLD	Large EV	0.720
[70]	2D-SWE	Cirrhosis	1-year death or liver transplantation	0.72 (*p* = 0.006)
[54]	p-SWE	Various etiologies	HP	0.712 (*p* = 0.010)
[70]	2D-SWE	Cirrhosis	1 year—liver decomposition	0.710 (*p* = 0.003)
[84]	ARFI	CHB cirrhosis	Medium EV	0.707 (95% CI 0.576–0.837)
[84]	2D-SWE-STE	CHB A2-3 inflammation activity	Cirrhosis	0.70 (95% CI 0.62–0.78, *p* < 0.51)
[69]	ARFI	Cirrhosis	HP	0.698 (*p* = 0.0274)
[84]	2D-SWE-STE	CHB, ALT > 2 Upper Normal Limit	Cirrhosis	0.67 (95% CI 0.57–0.76, *p* < 0.34)
[61]	2D-SWE-STE	CHB	S > 2 liver fibrosis	0.67 (95% CI 0.56–0.78)
[67]	ARFI	Compensated cirrhosis	HREV	0.66 (95% CI 0.62–0.70)
[84]	2D-SWE-STE	CHB	Cirrhosis	0.66 (95% CI 0.61–0.71, *p* < 0.001)
[84]	2D-SWE-STE	CHB, ALT < 2 Upper Normal Limit	Cirrhosis	0.66 (95% CI 0.60–0.71, *p* < 0.001)
[87]	2D-SWE	Patients after TIPS	Moderate gastroesophageal varices	0.655
[73]	TE (FibroScan)	CHC-related cirrhosis	Gastroesophageal varices	0.624
[87]	2D-SWE	Patients after TIPS	Mild gastroesophageal varices	0.585
[84]	2D-SWE-STE	CHB A0-1 inflammation activity	Cirrhosis	0.52 (95% CI 0.46–0.58, *p* < 0.001)

ALT: Alanine Aminotransferase; ARFI: Acoustic Radiation Force Impulse; AUROC: Area Under the Receiver Operating Characteristic Curve; CHB: Chronic Hepatitis B; CHC: Chronic Hepatitis C; CLD: Chronic Liver Disease; CSPH: Clinically Significant Portal Hypertension; ET: Essential Thrombocythemia; EV: Esophageal Varices; HREV: High-Risk Esophageal Varices; HP: Portal Hypertension (in the context of “Various etiologies HP”); MAFLD: Metabolic-Associated Fatty Liver Disease; MF: Myelofibrosis; MNs: Myeloproliferative Neoplasms; p-SWE: Point Shear Wave Elastography; PH: Portal Hypertension; PV: Polycythemia Vera; SSM: Spleen Stiffness Measurement; TE: Transient Elastography (FibroScan); TIPS: Transjugular Intrahepatic Portosystemic Shunt; VCTE: Vibration-Controlled Transient Elastography; VTQ: Virtual Touch Quantification; 2D-SWE: Two-Dimensional Shear Wave Elastography; 2D-SWE-STE: Two-Dimensional Shear Wave Elastography (Supersonic Shear Imaging variant).
